# Evaluation of Seaweed-Based Feed Additive on Enteric Methane Emissions of Grazing Heifers

**DOI:** 10.3390/ani15243625

**Published:** 2025-12-17

**Authors:** Jianwei Chen, Tamara L. Loiselle, Miles E. Theurer

**Affiliations:** 1 Synergraze Inc., Calgary, AB T2S 0C9, Canada; 2 Veterinary Research and Consulting Services LLC, Hays, KS 67601, USA

**Keywords:** bromoform, enteric emissions, feed additive, GreenFeed, pasture

## Abstract

This study evaluated the impact of a seaweed-based feed additive (SBFA) on enteric methane emissions in grazing heifers. Two groups of heifers (*n* = 11 per group) were maintained on tame pasture under identical conditions. The trial consisted of three phases: a two-week baseline period, a two-week adaptation period, and a seven-week full-dose period. During adaptation, the treatment group received SBFA once daily, with the dosage gradually increased to a target dose of 280 mg bromoform/head per day. This full dose was administered throughout the final phase. Enteric emissions of methane were continuously monitored using the GreenFeed emission monitoring system. During the baseline period, gas emissions were not different between the groups (*p* = 0.75); however, during the adaptation (*p* = 0.08) it tended to be lower in the SBFA group compared to the control, and during the full-dose period, methane emissions in the SBFA treatment group were significantly (*p* < 0.01) lower than in the control group (*p* < 0.01), averaging 53.7 g/d versus 203.2 g/d, corresponding to a 73.6% reduction in methane. Additionally, a prolonged suppression effect was observed, with methane emissions in the treatment group remaining low for three days after removal of the SBFA compared to the control group (*p* < 0.01), and on day 4 after the removal, the SBFA treatment group still tended (*p* = 0.07) to be lower than the control group. These findings indicate that SBFA, when administered once daily, has significant potential for mitigating enteric methane emissions in grazing cattle.

## Simple Summary

This study evaluated if a seaweed-based feed additive (SBFA) could reduce methane emissions in grazing heifers. Two groups of heifers (*n* = 11 per group) were maintained on pasture with one group of hand-fed SBFA and other control group. The trial consisted of three phases: a two-week baseline period, a two-week adaptation period, and a seven-week full-dose period. When administered at a full dose, the SBFA treatment group showed a significant reduction in methane emissions, averaging 73.6% lower than the control group (53.7 g/day vs. 203.2 g/day), and the results remained for up to four days after removal of the product. These findings indicate that SBFA, when administered once daily, has significant potential for mitigating enteric methane emissions in grazing cattle.

## 1. Introduction

Reducing enteric methane emissions from livestock is critical for mitigating climate change. Methane is a potent greenhouse gas (GHG) with a global warming potential 28–34 times greater than carbon dioxide over a shorter timeframe. Enteric methane emissions from ruminants, such as cattle and sheep, contribute approximately 39.1% of total livestock sector GHG emissions [1]. Various strategies have been explored to mitigate enteric methane emissions, including dietary modifications, such as incorporating feed additives like seaweed, 3-nitrooxypropanol (3-NOP), lipids, and selective breeding programs aimed at low-emissions ruminants [2,3]. Among these approaches, the inclusion of red seaweed, *Asparagopsis* spp., in livestock feed has emerged as a promising solution. Research indicates that even small quantities of *Asparagopsis* spp. using a seaweed-based feed additive (SBFA) can substantially reduce methane production in ruminants (Kinley et al., 2020 [4]), making it a highly effective and potentially transformative strategy for mitigating methane emissions. However, much of the *Asparagopsis* biomass used in animal trials comes from wild harvest, and large-scale cultivation of *Asparagopsis* spp. remains challenging, with only a few companies achieving limited commercial success [5,6]. Feeding small dosages of microalgae has been shown to have minimal impact on the shelf life of beef [7].

The methane-reducing properties of *Asparagopsis* spp. are primarily attributed to its high concentration of halogenated compounds, particularly bromoform [6]. While many seaweeds produce bromoform, only a few species accumulate it at significant levels. *Asparagopsis* spp. is the only species known to reach high enough bromoform (CHBr_3_) concentrations to be a viable feed additive for enteric methane mitigation. Upon ingestion, *Asparagopsis* releases bromoform into the rumen, where it effectively inhibits methanogens. Methanogens are the archaea which use the methanogenesis metabolic pathway responsible for methane production. Rumen methanogenesis combines carbon dioxide and hydrogen into methane and water. This inhibition targets methyl-coenzyme M reductase (MCR), a key enzyme in the final step of methanogenesis, thereby suppressing methane formation in the rumen [2].

Given the significant potential of *Asparagopsis* spp. for methane reduction and the challenges associated with its large-scale cultivation, alternative natural sources of bromoform accumulation are desirable. Recent research has introduced a method to enhance bromoform accumulation in fast-growing seaweeds such as kelp [8]. This multi-seaweed-based approach yields a product with sufficiently high bromoform concentrations, but did not include any *Asparagopsis* spp. Seaweed-based feed additive exhibited methane reductions comparable to or exceeding that of *Asparagopsis* spp., a widely studied SBFA derived from fast-growing seaweeds that can be cultivated at scale with fewer resource constraints, making it a promising alternative for commercial applications, particularly in large-scale livestock operations where a consistent and sustainable supply is essential.

The present study aims to evaluate the effectiveness of this novel product in reducing enteric methane emissions. Since grazing cattle contribute the highest methane emissions due to their fibrous forage-based diet, and limited research has focused on pasture-based systems, this study investigates the impact of the seaweed-based feed additive on grazing heifers in a tame pasture setting.

## 2. Materials and Methods

### 2.1. Animal Selection and Management

A total of 41 Angus crossbred heifers from the Olds College Smart Farm herd were initially enrolled in a training period to ensure that at least 24 animals would consistently utilize the GreenFeed emission monitoring (GEM; C-Lock, Inc., Rapid City, SD, USA) system. The selected heifers weighed between 454 and 544 kg, based on their most recent recorded weights from March 2024. Following selection, the heifers were moved to pasture for baseline data collection and subsequent methane emissions measurements under SBFA supplementation. Animal handling procedures were approved by the Olds College Animal Care and Use Committee. All heifers were monitored daily in both the feedlot pens and pastures for signs of illness, distress, or injury. None of the animals included in the official trial required any health treatments throughout the study period.

### 2.2. GreenFeed Emissions Monitoring Configuration

The GEM unit was first placed in feedlot pen for training. During this period, heifers were offered barley grain from the GEM feed bins to encourage voluntary visits. Access to the GEM unit was unrestricted. At the end of the 19-day training period (18 May to 5 June), 24 heifers were selected for the official trial based on their visit frequency. The total number of visits among selected heifers ranged from 109 to 315, with an average of 218 visits. The average number of daily visits per animal ranged from 5.7 to 16.6, with a mean of 11.5 visits per day.

For each animal visit, the GreenFeed system was configured to dispense a maximum of nine drops of oats, with a 20 s interval between drops and 30 g of feed per drop. Feed period was set to be 4 h with a maximum of six feed periods per day. The individual animal emissions rates for each day were averaged for all recorded feeding events (greater than 3 min in length with head in position). All calibrations were performed according to the manufacturer’s instructions. Daily environmental temperature and wind speed were captured with the GEM.

### 2.3. Baseline Data Collection

To start, the heifers from both the treatment and control group were moved to pastures as a single group, with access to both pastures. The same GEM unit was placed within a small enclosure accessible from both pastures. To encourage heifers to use the system, the GEM feed bins were stocked with oats. During the first week, each heifer received 1.3 kg of oats per day from concrete feed bunks. This daily allowance was subsequently reduced to 0.45 kg per head. During the baseline period, the heifers had partial access to both pastures. Baseline period lasted 2 weeks during study days −28 to −14.

### 2.4. Seaweed-Based Feed Additive Supplementation and Experimental Design

Prior to SBFA supplementation, the heifers were randomly assigned using random numbers from Excel to two groups (*n* = 12 per group). The treatment group and control group initially were placed into two separate fields with GEM access provided to each group on alternating days, but by week 2, the animals were all placed in a single field and shared GEM access simultaneously.

The novel SBFA used in this study was provided by Synergraze Inc. (Calgary, AB, Canada). This additive was formulated without the inclusion of *Asparagopsis* spp. and instead derived from a variety of fast-growing seaweed species [7]. The average bromoform concentration in the additive was 15 mg/g. Initially, SBFA was mixed in a bucket with 454 g of oats and hand-fed using buckets to individual heifers once daily. Early attempts at supplementation were unsuccessful, likely due to palatability issues. However, when supplementation was provided early in the morning (e.g., 7:00 a.m.), the heifers were more likely to consume the feed additive. Over time, the animals adapted to the supplementation, and for the remainder of the trial, all SBFA was provided in the early morning. Despite this adjustment, individual intake varied throughout the trial, with occasionally missed doses. The target SBFA dose was introduced gradually, beginning at 10% of the final intended dose and increasing over a two-week adaptation period until near-full intake was achieved (study day −14 to −1). Any unconsumed SBFA remaining in the bucket was manually collected and quantified to determine the actual intake per animal. Control group animals received an equivalent quantity of oats in feed bunks without the SBFA. During the adaptation period, one heifer in the SBFA group consistently refused to consume the supplement and was removed from the trial. To maintain balanced group sizes, an additional heifer was randomly removed from the control group, resulting in a final sample size of 11 heifers per group for a total of 22 heifers. Heifers were then fed the full dose for a period of 7 weeks from study day 0 to 49.

### 2.5. Residual Effects from Removal of Seaweed-Based Feed Additive

On study day 49, heifers in the SBFA group had the product removed. Both the control and SBFA group were monitored for an additional 7 days using the GEM equipment to determine the residual duration effect of the seaweed-based feed additive product.

### 2.6. Bromoform Measurement

Bromoform concentrations were analyzed using gas chromatography–mass spectrometry (GC 8890 and MS 5977C, Agilent, Santa Clara, CA, USA) with a headspace autosampler (HS 8697, Agilent, Santa Clara, CA, USA). The method was adapted from Franco et al. [9] with the following modifications:

- A 20 mL glass vial was used in the headspace autosampler and heated to 160 °C for 3 min. Nitrogen gas (Air Liquide Canada, Montreal, QC, Canada, 99.999% purity) was used to pressurize the vial.
- The gas chromatograph was equipped with a DB-624 column (UI 60 m × 0.32 mm × 1.80 µm) and operated with a split ratio of 20:1 with helium (Air Liquide Canada, Montreal, QC, Canada, 99.9999% purity) as the carrying gas.
- The oven temperature was programmed as follows: initial hold at 40 °C for 1 min, followed by an increase to 200 °C at a rate of 30 °C/min, with a final hold for 4 min.
- The mass spectrometer operated with a selected ion monitoring (SIM) mode at *m*/*z* 173 and 252, with a dwell time of 50 ms per ion.

### 2.7. Statistical Analysis

Data were evaluated using a commercial software program (rStudio Team version 4.5.2, 2025, Boston, MA, USA). Environmental outcomes (methane and carbon dioxide emissions) were evaluated as a treatment by time interaction including random effect for repeated measures on the individual animal. Environmental outcomes were also evaluated with treatment by time period (baseline, adaptation, and full dose) including random effect for repeated measures on individual animal. Methane emissions after removal of the seaweed-based feed additive were evaluated as a treatment by time interaction including random effect for repeated measures on the individual animal. Pairwise comparisons between treatment groups within days since the removal were performed. Differences exhibiting a *p* value ≤ 0.05 were considered statistically significant with tendencies described as *p* > 0.05 and ≤0.10 for all outcomes evaluated.

## 3. Results

Minimum, maximum, and average environmental temperatures and wind speed are displayed in Table 1. There was a treatment group-by-time period interaction for methane (*p* < 0.01) and carbon dioxide emissions (*p* = 0.02; Table 2). During the baseline period, there were no differences between treatment groups (*p* > 0.70). During the 2-week adaptation period (study day −14 to −1), heifers in the SBFA group tended to have lower methane emissions compared to the control group (*p* = 0.08). The adaptation period began on day −14. The seaweed-based feed additive was introduced at a low dose to the treatment group to initiate the formal trial. Initially, many treatment heifers were reluctant to consume the supplement. Over time, most heifers gradually accepted it, though not all. By the end of the 2-week adaptation period, most heifers reached nearly a full dose.

The full-dose time period, during which a fixed daily supplement dose of 280 mg/head of SBFA was provided, ran from study day 0 to 49. The average bromoform intake was 18.6 g/head/day which was slightly higher than the targeted 15 g/head/day. While most treatment heifers consumed the majority of the supplement, occasional refusals occurred, and some animals missed one or two full-day doses. The average daily bromoform intake, calculated based on leftover supplement, remained relatively stable at 237 mg/head. Overall, the SBFA treatment group achieved 77.6% of the target dose.

**Figure 1:**
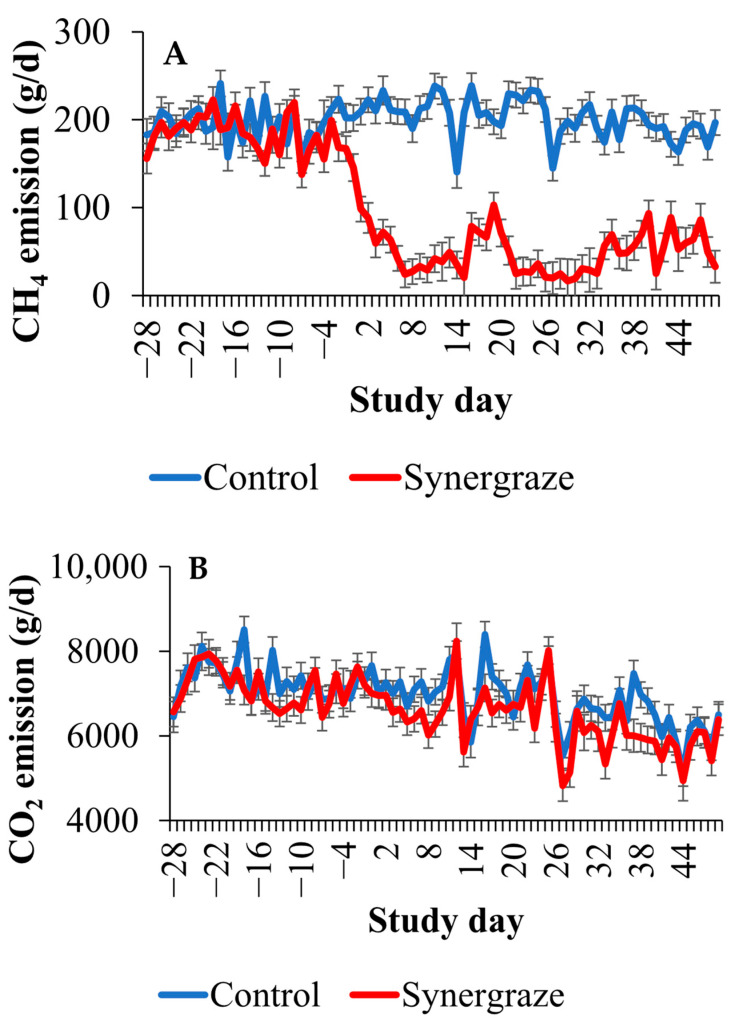
Model-adjusted daily methane (**A**) and carbon dioxide (**B**) emissions by treatment group (*n* = 11 control and *n* = 11 seaweed-based feed additive) and study day. Control treatment group represented by blue line and seaweed-based feed additive treatment group represented by red line. Study days −28 to −15 are the baseline period, days −14 to −1 are the adaptation period, and 0 to 49 are the full-dose period. Model included fixed effect of study day, treatment group, interaction between treatment group and trial day with random effects for repeated measures on the individual animal. Treatment group by time was statistically significant (*p* < 0.01) for both models.

**Figure 2:**
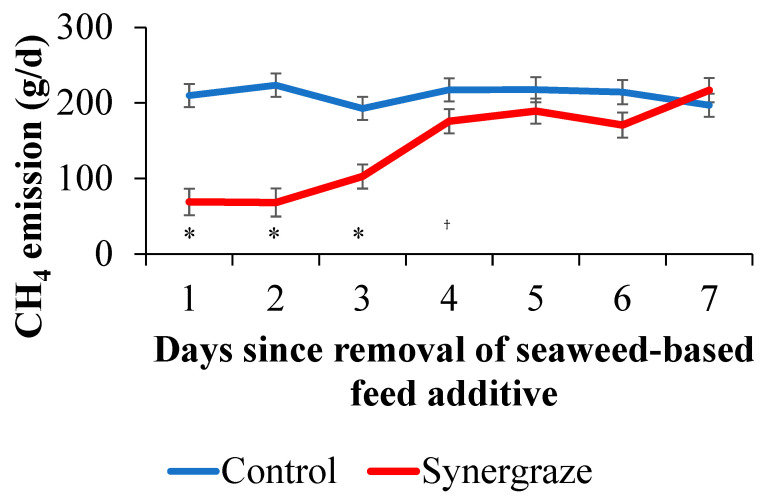
Model-adjusted daily methane emission by treatment group (*n* = 11 control and *n* = 11 seaweed-based feed additive) and days since removal of the seaweed-based feed additive. Control treatment group represented by blue line and seaweed-based feed additive treatment group represented by red line. Model included fixed effect of study day, treatment group, interaction between treatment group and trial day with random effects for repeated measures on the individual animal. Significant differences (*p* < 0.01) noted with * or (*p* = 0.07) noted with ^†^ between treatment group within days since removal.

As shown in Figure 1, methane emissions in the SBFA treatment group began declining during the adaptation period and remained lower throughout the full-dose period. Minimal differences between treatment groups were observed for carbon dioxide emissions. During the full-dose period, the SBFA treatment group had 73.6% lower methane emissions compared to the control group (*p* < 0.01). Following the cessation of SBFA supplementation, GEM monitoring continued for an additional week to evaluate the recovery of methane emissions in the treatment group. As shown in Figure 2, methane emissions remained low immediately after supplementation ended. No significant increase was observed for the first 2 days (*p* < 0.01). Three days after the removal of SBFA, the SBFA treatment group still had significantly lower methane emissions compared to the control group (*p* < 0.01), and on day 4 after the removal, the SBFA treatment group still tended (*p* = 0.07) to be lower than the control group.

## 4. Discussion

The two-month trial of grazing heifers on tame pasture supplemented with SBFA demonstrated a significant reduction in enteric methane emissions. The findings of this study demonstrate that the novel SBFA has the potential to substantially reduce enteric methane emissions in grazing cattle. Previous research on bromoform-containing seaweed has primarily focused on *Asparagopsis* spp., as it is currently the only known species with sufficiently high bromoform concentrations. Depending on diet composition and dosage, inclusion of *Asparagopsis* biomass in livestock feed has been shown to reduce CH_4_ emissions by 26.8–98% [4,10,11,12]. Some other alternative seaweed species mainly with different potential anti-methanogenic inhibitors have also shown a comparable methane inhibition level, but usually requires a significant inclusion rate [12]. This indicates bromoform as one of the most competitive seaweed bioactives for enteric methane mitigation. Responses in the SBFA treatment group may also be related to other bioactives or synergistic compounds contained in the product.

Eason and Fennessy [13] reported a dose-dependent relationship between bromoform intake and CH_4_ reduction. Based on this relationship, and assuming a daily dry matter intake (DMI) of 10 kg for the current study, the SBFA provided bromoform at a lower-end dosage of approximately 23 mg/kg of DMI. However, the SBFA achieved a high-end CH_4_ reduction of 15.2 g/kg of DMI dosage compared to the 6 g/kg of DMI predicted by the model cited in Eason’s study which was related to bromoform intake and CH_4_ reduction [13]. Conversely, the equivalent methane reduction of 15 g/kg dosage as indicated on the model was achieved with a 60 mg/kg DMI bromoform dosage. These findings suggest that SBFA could be a viable alternative to *Asparagopsis* for enteric methane mitigation and further supports bromoform as the primary functional compound.

Bromoform has probable carcinogenic and ozone-depleting properties at high concentrations [12,14]. Previous work has evaluated that 70% of CHBr_3_ was degraded within the first 30 min and 90% within the first 3 h [15]. The bromoform goes through rapid dehalogenation with a half-life of 26 min in the rumen [16]. Sunflower oil enriched with CHBr_3_ from *Asparagopsis taxiformis* showed no CHBr_3_ residues in the meat [17]. Bromoform meat residues have not been evaluated with the current SBFA product in the current study.

The residual effect of the seaweed-based feed additive lasted up to 4 days after removal of the SBFA. In the current study, heifers were administered the SBFA daily; however, the prolonged reduction in methane emissions for 4 days after removal may indicate the opportunity to administer it less frequently while retaining efficacy. The mechanism underlying this prolonged inhibition remains unclear and additional research is needed. Endurability of methane reduction is another critical factor when evaluating anti-methanogenic strategies. Endurability can be assessed in two ways: short-term (within hours of a single dose) and long-term (over weeks or months). Once ingested, bioactive compounds are released into the rumen and simultaneously begin to degrade. In vitro studies have shown that 70% of bromoform degrades within the first 30 min [15], suggesting that frequent supplementation (e.g., twice daily) may be necessary to maintain methane suppression. Other methane inhibitors, such as 3-NOP demonstrates approximately 28% methane reduction, but only for two and a half hours after ingestion [18]. The SBFA did not exhibit the same pattern as the impact of methane reduction lasted up to 4 days after removal of the supplement. Further evaluation of DMI is needed during the removal period.

Using the GEM to capture emissions data required training the heifers to utilize the equipment. The potential of heifers utilizing the GEM more frequently may provide selection bias; however, the heifers in both treatment groups were selected from the larger group which had appropriate visit frequency prior to trial beginning. While the GEM was only able to quantify intermittent emissions data, studies comparing the GEM to other methods to quantify cattle emissions data have been previously reported with high accuracies [19,20,21].

Despite these promising results, a key limitation of the present study is its relatively short duration. While significant methane reduction was confirmed over seven weeks, long-term studies are needed to assess inhibition over extended periods. Additionally, further improvements in palatability may be necessary to maximize intake and ensure consistent methane inhibition across the herd, as not all animals consistently consumed the full dose. Optimizing formulation to enhance texture and taste could improve voluntary intake and overall efficacy. Removing one heifer due to its refusal to consume the oats with the SBFA and then randomly removing one control heifer reduced statistical power, which was part of the reason of using *p* ≤ 0.10 as the significance threshold knowing that there was an increased chance of type I error. Additionally, the palatability of the SBFA product was initially challenging; however, feeding the SBFA only during the first feeding when heifers were more aggressive to the feed helped overcome the palatability issue.

## 5. Conclusions

This study demonstrated that the novel SBFA significantly reduces enteric methane emissions in grazing heifers. Over the seven-week period, SBFA supplementation achieved an average methane reduction of 73.6%. Notably, a prolonged inhibition effect persisted for up to 4 days after supplementation ceased, suggesting that SBFA may sustain its anti-methanogenic efficacy beyond immediate ingestion. The observed reduction in methane emissions highlights the feasibility of incorporating SBFA into pasture-based systems, addressing a critical gap in current methane mitigation strategies. The fact that the sustained inhibition effect was observed for 4 days upon removal, even with once-daily supplementation, suggests that SBFA could offer practical benefits for on-farm implementation, where frequent dosing is logistically challenging. These findings highlight SBFA as a promising alternative to *Asparagopsis* spp. for enteric methane mitigation. Its effectiveness with once-daily supplementation enhances its practicality for real-world implementation, particularly in pasture-based systems; however, further research is needed to assess its long-term efficacy, improve palatability, and elucidate its effects on rumen microbial communities to optimize its application in large-scale livestock production.

## Figures and Tables

**Table 1:** Minimum, maximum, and average environmental temperatures and wind speed by study day and period.

Study Day	Period	Min Temperature (°C)	Max Temperature (°C)	Avg Temperature (°C)	Min Wind Speed (m/s)	Max Wind Speed (m/s)	Avg Wind Speed (m/s)
−28	Baseline	9.17	26.12	17.60	0.20	2.83	0.98
−27	Baseline	18.49	28.68	25.04	0.32	1.73	0.79
−26	Baseline	13.31	32.97	20.63	0.19	1.71	0.60
−25	Baseline	10.87	21.25	13.85	0.22	1.83	0.84
−24	Baseline	7.48	15.39	12.02	0.87	2.71	1.77
−23	Baseline	6.65	19.70	13.42	0.12	1.80	0.98
−22	Baseline	4.87	19.58	13.32	0.13	1.09	0.54
−21	Baseline	4.64	23.98	16.66	0.02	0.92	0.41
−20	Baseline	7.19	28.32	15.26	0.15	1.10	0.49
−19	Baseline	10.81	28.90	21.62	0.07	1.35	0.80
−18	Baseline	9.24	29.93	20.17	0.00	1.34	0.65
−17	Baseline	14.89	25.46	19.72	0.14	0.87	0.46
−16	Baseline	11.08	28.91	22.44	0.23	1.03	0.59
−15	Baseline	10.74	25.42	19.67	0.00	2.44	1.21
−14	Adaptation	10.41	28.78	19.59	0.03	1.88	0.92
−13	Adaptation	12.62	17.37	15.04	0.00	2.00	0.82
−12	Adaptation	12.40	25.77	19.33	0.00	2.20	1.19
−11	Adaptation	9.63	29.24	19.91	0.00	1.28	0.63
−10	Adaptation	14.02	21.56	17.27	0.05	1.08	0.65
−9	Adaptation	14.65	27.83	18.91	0.35	1.84	1.05
−8	Adaptation	14.19	30.32	20.01	0.21	1.63	0.79
−7	Adaptation	12.47	16.62	13.48	0.67	2.32	1.80
−6	Adaptation	14.05	29.19	20.83	0.08	1.27	0.71
−5	Adaptation	12.75	23.48	18.54	0.37	1.46	0.80
−4	Adaptation	14.81	29.29	21.35	0.00	1.50	0.86
−3	Adaptation	13.74	37.78	24.52	0.00	1.27	0.39
−2	Adaptation	16.06	39.24	27.11	0.01	1.20	0.53
−1	Adaptation	18.07	40.60	26.22	0.00	1.23	0.47
0	Full dose	17.87	35.63	24.28	0.19	0.69	0.36
1	Full dose	17.06	29.63	23.20	0.28	2.40	1.34
2	Full dose	17.69	29.81	20.72	0.23	1.66	0.73
3	Full dose	14.73	36.32	25.26	0.00	1.13	0.46
4	Full dose	15.24	27.82	21.13	0.00	0.64	0.20
5	Full dose	16.79	37.39	25.76	0.00	0.98	0.38
6	Full dose	19.00	40.03	28.10	0.04	1.08	0.45
7	Full dose	17.90	41.35	27.31	0.00	0.88	0.20
8	Full dose	16.48	42.12	27.08	0.00	1.56	0.44
9	Full dose	18.47	42.26	29.03	0.00	2.08	0.47
10	Full dose	18.76	36.65	28.27	0.00	1.59	0.58
11	Full dose	18.57	46.67	30.31	0.00	0.83	0.43
12	Full dose	17.73	35.98	27.62	0.15	0.83	0.39
13	Full dose	24.14	37.04	31.30	0.11	1.86	0.85
14	Full dose	20.77	36.93	26.40	0.25	1.75	1.06
15	Full dose	16.24	21.82	19.69	0.27	2.28	1.47
16	Full dose	15.13	25.52	19.39	0.21	1.41	0.67
17	Full dose	12.88	28.39	19.21	0.28	1.37	0.60
18	Full dose	13.99	36.62	23.13	0.00	1.40	0.68
19	Full dose	13.16	40.86	22.84	0.18	2.52	0.57
20	Full dose	16.08	37.77	23.67	0.01	0.77	0.35
21	Full dose	14.94	41.29	28.41	0.23	1.93	0.66
22	Full dose	13.77	38.55	24.46	0.00	1.44	0.51
23	Full dose	18.43	32.50	25.39	0.15	2.16	1.10
24	Full dose	14.26	26.94	19.43	0.19	1.47	0.59
25	Full dose	14.64	33.52	22.08	0.07	1.26	0.56
26	Full dose	15.89	28.08	19.31	0.22	2.18	1.14
27	Full dose	15.43	17.63	16.24	0.18	0.98	0.56
28	Full dose	14.67	30.14	19.03	0.12	1.34	0.72
29	Full dose	10.52	28.15	18.99	0.02	0.85	0.38
30	Full dose	23.37	29.85	25.94	0.55	1.04	0.85
31	Full dose	17.49	33.00	28.45	0.00	1.18	0.80
32	Full dose	11.69	36.73	23.93	0.00	1.08	0.35
33	Full dose	13.95	32.65	22.41	0.15	2.32	0.81
34	Full dose	12.63	32.69	21.55	0.01	1.40	0.49
35	Full dose	15.12	32.48	21.73	0.00	1.40	0.63
36	Full dose	15.36	30.25	22.79	0.00	2.24	1.04
37	Full dose	16.02	22.52	18.95	0.00	1.32	0.50
38	Full dose	15.16	28.92	20.44	0.00	1.42	0.69
39	Full dose	13.64	33.30	22.28	0.00	1.61	0.52
40	Full dose	14.79	37.52	24.75	0.06	1.96	0.62
41	Full dose	16.78	30.30	22.83	0.19	2.40	0.91
42	Full dose	15.24	21.69	17.56	0.06	1.66	0.71
43	Full dose	13.70	26.48	19.41	0.10	1.54	0.89
44	Full dose	21.29	30.04	26.87	0.51	1.64	1.06
45	Full dose	13.47	30.01	20.17	0.03	3.15	1.56
46	Full dose	11.82	31.77	20.32	0.11	1.28	0.63
47	Full dose	10.45	30.47	20.19	0.09	1.82	0.82
48	Full dose	12.57	27.08	20.29	0.49	3.22	1.55
49	Full dose	10.70	13.87	12.18	1.25	2.81	2.07
50	Removal	11.14	33.23	22.41	0.28	1.48	0.88
51	Removal	10.09	35.77	20.04	0.02	2.14	1.11
52	Removal	11.44	34.96	21.85	0.00	1.44	0.54
53	Removal	11.66	40.34	25.05	0.00	1.38	0.49
54	Removal	15.64	36.00	21.49	0.03	1.72	1.08
55	Removal	16.45	38.42	27.15	0.00	1.19	0.57
56	Removal	13.26	21.65	17.26	0.07	1.45	0.65

**Table 2:** Model-adjusted least square means (±SE) of environmental outcomes by treatment group (*n* = 11 control and *n* = 11 seaweed-based feed additive) and period through 49 days on feed. Models included fixed effect of treatment group, period, treatment group-by-period interaction, and random effect for repeated measures on individual animal. Treatment group-by-period was significant for methane (*p* < 0.01) and carbon dioxide (*p* = 0.02).

Period	Outcome	Control	Seaweed-Based Feed Additive	SE	*p* Value
Baseline	Methane, g/d	197.3	193.6	8.12	0.75
	Carbon dioxide, g/d	7449.8	7351.3	222.15	0.76
Adaptation	Methane, g/d	195.8	175.1	8.07	0.08
	Carbon dioxide, g/d	7189.9	6946.6	221.46	0.44
Full dose	Methane, g/d	203.2	53.7	7.36	<0.01
	Carbon dioxide, g/d	6802.7	6370.4	211.57	0.16

## Data Availability

The data presented in this study are available on request from the corresponding author.

## Abbreviations

The following abbreviations are used in this manuscript:

MCR	Methyl-coenzyme M reductase
SBFA	Seaweed-based feed additive
GHG	Greenhouse gas
GEM	GreenFeed emission monitoring
CH_4_	Methane
3-NOP	3-nitrooxypropanol
CHBr_3_	Bromoform
